# Prediction of metabolic dysfunction-associated steatotic liver disease among patients with type 2 diabetes mellitus using liver fat indices in a resource-limited clinical setting

**DOI:** 10.3389/fcdhc.2026.1916533

**Published:** 2026-09-25

**Authors:** KKDSD Kannangara, WAC Karunarathne, NRP Perera, Uditha Bulugahapitiya, JMKB Jayasekara, Nimantha Karunathilaka

**Affiliations:** 1 Faculty of Graduate Studies, General Sir John Kotelawala Defence University, Rathmalana, Sri Lanka; 2 Department of Medical Laboratory Science, Faculty of Allied Health Sciences, General Sir John Kotelawala Defence University, Werahera, Sri Lanka; 3 Faculty of Allied Health Sciences, General Sir John Kotelawala Defence University, Werahera, Sri Lanka; 4 Diabetic and Endocrinology Unit, National Hospital, Colombo, Sri Lanka; 5 Department of Nursing and Midwifery, Faculty of Allied Health Sciences, General Sir John Kotelawala Defence University, Werahera, Sri Lanka

**Keywords:** liver fat indices, metabolic dysfunction –associated steatotic liver disease, non-alcoholic fatty liver disease, type 2 diabetes mellitus, ultrasonography

## Abstract

**Introduction:**

Metabolic dysfunction-associated steatotic liver disease (MASLD), previously termed non-alcoholic fatty liver disease (NAFLD), is the hepatic presentation of metabolic syndrome. It shares the common pathophysiology of insulin resistance with type 2 diabetic mellitus (T2DM). The aim of this study was to establish the predictive ability of non-invasive liver fat indices—Fatty Liver Index (FLI), Hepatic Steatosis Index (HSI), Visceral Adiposity Index (VAI), Triglyceride and Glucose Index (TyG), and NAFLD-Liver Fat Score (NAFLD-LFS)—to determine fatty liver among patients with T2DM.

**Methodology:**

A cross-sectional analytical study was conducted on 260 newly diagnosed patients with type 2 diabetes. Anthropometric measurements were taken, while ultrasound scan was performed to determine fatty liver; its grade and blood samples were used to determine biochemical parameters. Liver fat index scores were calculated using standardized equations and analyzed using the statistical software SPSS version 23.0.

**Results:**

The incidence of MASLD among patients with T2DM was 69.2%. Three of the liver fat indices—FLI, HSI, and NAFLD-LFS—showed strong statistically significant correlations (*p* < 0.001) with liver ultrasonographic findings while the remaining indices—VAI and TyG—showed moderately significant correlations (*p* < 0.01). The cutoff values, sensitivities, and specificities of each index were determined by receiver operating characteristic (ROC) curve analysis. FLI showed the highest diagnostic performance [area under the curve (AUC) = 0.947; 95% confidence interval (CI): 0.923–0.971] with 75% sensitivity and 100% specificity with a cutoff value of 46.12. Both HSI (AUC = 0.847; 95% CI: 0.812–0.902) and NAFLD-LFS (AUC = 0.847; 95% CI: 0.800–0.894) showed good diagnostic performance with sensitivities of 68.3% and 76.1%, specificities of 95.5% and 85%, and cutoff values of 37.02 and 0.906, respectively. A moderate diagnostic performance was observed in the remaining indices: TyG (AUC = 0.707; 95% CI: 0.642–0.772) and VAI (AUC = 0.670; 95% CI: 0.602–0.739). Based on the multivariable logistic regression analysis, both FLI [adjusted odds ratio (aOR) = 1.144, 95% CI: 1.090–1.201, *p* ≤ 0.001] and HSI (aOR = 1.366, 95% CI: 1.157–1.614, *p* ≤ 0.001) were able to independently predict the presence or absence of fatty liver.

**Conclusion:**

The current study showed that all five liver fat indices have the ability to predict the presence or absence of fatty liver among patients with T2DM. FLI and HSI showed the highest performance among the five indices, which can be used as screening tools for fatty liver more efficiently. The findings provide reliable alternative methods for detecting fatty liver in a resource-limited clinical setting simplifying patient management.

## 1 Introduction

Metabolic dysfunction-associated steatotic liver disease (MASLD), formerly known as non-alcoholic fatty liver disease (NAFLD), is the most common chronic liver disease worldwide and is in continuous rise (1). It is defined as the presence of hepatic steatosis, which is the accumulation of fat in liver hepatocytes exceeding 5%–10% of liver weight accompanied by at least one of the cardio-metabolic risk factors and without alcohol or limited alcohol consumption (2).

Diabetes mellitus is a group of metabolic disorders characterized by hyperglycemia that negatively affects insulin secretion, insulin action, or both. Diabetes can be classified into several groups according to mode of action and consists of type 1 diabetes mellitus (T1DM), type 2 diabetes mellitus (T2DM), gestational diabetes mellitus (GDM), and other specific types of diabetes mellitus (3).

MASLD is strongly linked to T2DM as both conditions are related to metabolic syndrome and share the common pathophysiology of insulin resistance. The co-occurrence of MASLD and T2DM is considerably high and exceeds 70%, which further leads to more health complications for patients (4).

Liver biopsy is the gold standard in diagnosing fatty liver, but it is an invasive and costly procedure and can cause unfavorable outcomes to the patient (5). The most common technique used at present is ultrasonography. Many other radiological approaches are also available like magnetic resonance imaging (MRI) and computed topography (CT). In recent years, transient elastography (FibroScan) has been introduced to measure fat accumulation and liver stiffness, which aids in the diagnosis of fatty liver as an alternative method to liver biopsy (6) but is not widely practiced due to the unavailability of resources and its expensive nature. Therefore, developing simple, routine biomarker panels and validating the existing liver fat indices according to the requirements of the local population are important to easily predict MASLD/NAFLD to reduce the disease burden.

Liver fat indices, which involve non-invasive procedures, can be used to predict MASLD. The indices used in this study are Fatty Liver Index (FLI) (7), Hepatic Steatosis Index (HSI) (8), Visceral Adiposity Index (VAI) (9), Triglyceride and Glucose Index (TyG) (10), and NAFLD-Liver Fat Score (NAFLD-LFS) (11). The use of liver fat indices would be more cost-effective as it mostly includes routinely carried out laboratory investigations and several anthropometric measurements that could be easily accessed in the Sri Lankan clinical setup. Therefore, the aim of this study was to determine the association of the liver ultrasonography-based diagnosis of fatty liver with the five liver fat indices proposed to introduce a worthwhile method of predicting fatty liver and refer vulnerable patients at an early stage for further treatments and lifestyle modifications, leading to fewer complications.

Based on the recent international consensus shifting from NAFLD to MASLD, the current study analysis has been aligned to the MASLD classification to ensure its clinical relevance. However, the research data produced before the recent consensus establishment is referred to as NAFLD in this context.

## 2 Methods

### 2.1 Ethical considerations

Ethical clearance was obtained from the General Sir John Kotelawala Defence University on 30 April 2021 (RP/2020/27).

Written consent was obtained from all participants, and all data collected from the participants were handled while maintaining confidentiality.

### 2.2 Study design

A cross-sectional analytical study was conducted on newly diagnosed type 2 diabetic patients for a period of 2 years.

### 2.3 Study setting/population

A total of 260 newly diagnosed patients with type 2 diabetes who were registered at the diabetic and endocrinology clinic at the Colombo South Teaching Hospital, Kalubowila from January 2022 to January 2024 were selected based on inclusion and exclusion criteria. The diagnosis of patients with T2DM were confirmed by a consultant endocrinologist using fasting plasma glucose levels, postprandial blood glucose levels, and medical history (12). All the procedures of the study were carried out according to the principles of the Declaration of Helsinki (13). An informed consent was obtained from the patients, and those who did not provide consent were excluded from the study.

The absence of or limited alcohol consumption was considered in selecting individuals for the study following the AASLD guidelines (14) where it is defined to be <20 g/day in women and <30 g/day in men. Patients who are above 65 years, pregnant women, patients with T1DM, and patients with chronic liver diseases, endocrine disorders, hepatic viral infections, and autoimmune disorders were excluded from the study. Individuals who started treatment for T2DM were also excluded from the study.

The patients who previously met the NAFLD criteria overlap with MASLD diagnostic criteria based on the fulfillment of at least one of the risk factors of metabolic syndrome.

### 2.4 Data collection methods and tools

An information sheet was provided with regard to the research project, and all participants were informed to fast for 10–12 h to collect blood for biochemical investigations.

An interviewer-administered questionnaire was conducted for the participants to gather socio-demographic data, family and clinical history, and necessary anthropometric measurements. The data collected included age, gender, height, weight, waist circumference, frequency of alcohol consumption, smoking history, and body mass index (BMI).

Blood (5 mL) was drawn from each participant and added to both plain tubes and sugar tubes (sodium fluoride/potassium oxalate) to perform biochemical investigations. The samples were centrifuged, and serum and plasma were separated and stored at −80°C until the tests were performed.

An ultrasound scan of each participant was conducted by the consultant radiologist to diagnose and determine the stage of fatty liver using the Toshiba APLIO 500 Ultrasound system (Toshiba America Medical Systems, Inc., 2441 Michelle Drive, Tustin, CA, USA). The relevant measurements and observations were made by placing the probe on the sagittal plane along the mid-auxiliary line, and the presence or absence of fatty liver was determined based on the echogenicity of the liver. The grade of fatty liver was determined based on the degree of echogenicity and visualization of liver (15).

The five liver fat indices were calculated with the respective anthropometric details and the biochemical investigations using Equations 1–5.

- Fatty Liver Index (FLI).
  (1) $$\text{FLI}=({\text{e}}^{0.953\times \text{loge}(\text{triglycerides})+0.139\times \text{BMI}+0.718\times \text{loge}(\text{GGT})+0.053\times \text{waist circumference}-15.745})/(1+{\text{e}}^{0.953\times \text{loge}(\text{triglycerides})+0.139\times \text{BMI}+0.718\times \text{loge }(\text{GGT})+0.053\times \text{waist circumference}-15.7\;45})\times 100$$

- Hepatic Steatosis Index (HSI).
  (2) HSI=8×(ALT/AST)+BMI+2, if T2DM;+2, if female

- Visceral Adiposity Index (VAI).
  (3-I) Men:VAI=[Waist circumference/39.68+(1.88×BMI)]×[Triglycerides(TG)/1.03]×(1.31/HDL)
  (3-II) Women:VAI=[Waist circumference/36.58+(1.89×BMI)]×(TG/0.81)×(1.52/HDL)

- Triglyceride and Glucose Index (TyG).
  (4) TyG=log[Triglyceride(TG)×Fasting Blood sugar(FBS)/2]

- NAFLD-Liver Fat Score (NAFLD-LFS).
  (5) NAFLD−LFS=−2.89+1.18×metabolic syndrome(yes=1/no=0)+0.45×T2DM(yes=2/no=0)+015×Fasting serum insulin+0.04×AST−0.94×AST/ALT

### 2.5 Laboratory analysis

All the biochemical investigations were conducted at the laboratory of the Faculty of Allied Health Sciences, KDU within 1 week of sample collection. All biochemical investigations were conducted using a semi-automated analyzer—BTS-350 (Biosystems, Costa, Brava 30, 08030 Barcelona, Spain)—under the supervision of the supervisors and medical laboratory technologists. The biochemical investigations were conducted to determine alanine aminotransferase (ALT), aspartate aminotransferase (AST), gamma-glutamyl transferase (GGT) and serum triglyceride levels, high-density lipoprotein (HDL) levels, and fasting blood sugar (FBS). Fasting serum insulin levels were conducted at a referral laboratory using a fully automated analyzer—Roche Cobas E411 (Roche Diagnostics GmbH, Mannheim, Germany).

### 2.6 Data analysis

All the data collected (socio-demographic data, anthropometric measurements, results of biochemical investigations, results of ultrasonographic findings, and the scores obtained for each index) were entered into the statistical software Statistical Package for Social Sciences (SPSS) version 23.0 (IBM Corp., Armonk, New York, USA).

The study group of the research consisted of patients with type 2 diabetes diagnosed with NAFLD, and the control group included patients with type 2 diabetes without fatty liver.

All the data were summarized using descriptive statistics to obtain the basic information on the study population of the current research.

Some variables were further classified to aid in the data analysis to achieve the objectives of the study. BMI was calculated using the data collected and grouped based on the Asian categorization of BMI (16). The classification was as follows: underweight (BMI < 18.5), normal weight (BMI: 18.5–22.9), overweight (BMI: 23–24.9), pre-obese (25–29.9), and obese (BMI ≥ 30).

Waist circumference was also recorded and classified based on the gender for the South Asian population, where the waist circumference for men was approximated as 90 cm (35.5 inches) and 80 cm (31.5 inches) for women. The classification was based on the International Diabetic Federation (17).

Several statistical approaches like Pearson correlations, chi-square tests, independent samples *t*-test, effect size, area under the curve (AUC), and receiver operating characteristic (ROC) curve were used to obtain the final outcomes of the research study. Also, multivariable logistic regression was performed to determine the independent predictive ability of each index against the presence or absence of fatty liver after the adjustment of control variables (age, gender, BMI, waist circumference, FBS, HDL, triglyceride level, ALT, AST, GGT, and insulin) based on each index. The evaluation was done separately on each index excluding the demographic and biochemical variables that are not included in the specific index’s formula to prevent multicollinearity. The results were reported as adjusted odds ratio (aOR) with 95% confidence intervals (CIs). Multicollinearity was assessed using variance inflation factors (VIFs) to determine whether all values fall below the standard threshold value of 5.0. A *p*-value < 0.05 was considered statistically significant.

## 3 Results

A total of 260 newly diagnosed patients with type 2 diabetes were included in the study. Majority of the participants were women, which comprised 67% (*n* = 173) of the study population. All the variables used in this research study were following normal distribution.

The participants were between the ages of 25 and 65 years, and the statistical analysis showed that majority of the participants were between the age ranges of 41 and 55 years, which was 45.8% (*n* = 119) of the total study population. Thirty percent (*n* = 78) of the participants were ≥56 years of age and 24.2% (*n* = 63) were between 25 and 40 years of age.

Majority of the participants were diagnosed with fatty liver through liver ultrasonography, which comprised 69.2% (*n* = 180) of the study population. The classification based on the grade of fatty liver showed that 23.4% (*n* = 61) of participants had grade 1 fatty liver while 45.8% (*n* = 119) of participants were diagnosed with grade 2 fatty liver or above.

Out of the female patients, 69.9% (*n* = 121/173) were diagnosed with fatty liver based on the radiological investigations. The incidence of fatty liver was similar among male patients, with 67.8% (*n* = 59/87) diagnosed with fatty liver.

When considering the fatty liver diagnosis based on age, majority of the patients diagnosed with a fatty liver were between the age of 41 and 55 years, which comprised 50% (*n* = 90) of the total participants diagnosed with a fatty liver. Participants above ≥56 years showed an incidence of 27.2% (*n* = 49), while the remainder—those between the age of 25 and 40 years—showed an incidence of 22.8% (*n* = 41).

The descriptive statistics were conducted based on the gender to get a clear understanding of the variation in results between the female and male population of the study (**Table 1**) as well as based on the presence or absence of fatty liver (**Table 2**).

**Table 1:** Descriptive statistics based on gender.

Characteristics	Mean ± SD	Gender	
		Male (mean ± SD)	Female (mean ± SD)
Age (years)	48.23 ± 10.70	47.62 ± 10.89	48.54 ± 10.63
Weight (kg)	64.611 ± 11.16	68.03 ± 12.03	62.89 ± 10.32
Height (cm)	157.931 ± 8.46	164.30 ± 7.80	154.73 ± 6.82
BMI	25.89 ± 3.86	25.14 ± 3.56	26.27 ± 3.95
Waist (cm)	89.96 ± 8.68	89.68 ± 8.61	90.09 ± 8.74
FBS (mg/dL)	166.08 ± 57.57	161.03 ± 48.72	168.62 ± 61.52
HDL (mg/dL)	47.10 ± 6.55	46.77 ± 3.36	47.27 ± 6.66
Triglyceride (mg/dL)	154.65 ± 50.25	158.75 ± 46.93	152.60 ± 51.85
AST (U/L)	36.05 ± 17.51	39.36 ± 17.91	34.39 ± 17.12
ALT (U/L)	35.94 ± 18.89	38.89 ± 19.54	34.46 ± 18.42
GGT (U/L)	45.78 ± 23.75	48.46 ± 23.67	44.43 ± 23.75
Insulin	12.81 ± 5.66	11.57 ± 4.80	13.44 ± 5.96
HSI	37.22 ± 4.36	35.03 ± 3.83	38.33 ± 4.21
FLI	50.04 ± 24.70	49.33 ± 23.04	50.40 ± 25.56
TyG	5.02 ± 0.27	5.03 ± 0.25	5.02 ± 0.28
VAI	2.53 ± 1.04	1.20 ± 0.73	2.81 ± 1.07
NAFLD-LFS	1.49 ± 1.39	1.30 ± 1.31	1.59 ± 1.42

BMI, body mass index; FBS, fasting blood sugar; HDL, high-density lipoproteins; AST, aspartate aminotransferase; ALT, alanine aminotransferase; GGT, gamma-glutamyl transferase; HSI, Hepatic Steatosis Index; FLI, Fatty Liver Index; TyG, Triglyceride and Glucose Index; VAI, Visceral Adiposity Index; NAFLD-LFS, NAFLD-Liver Fat Score.

**Table 2:** Descriptive statistics based on the presence/absence of fatty liver.

Characteristics	Mean ± SD	Liver ultrasonography	
		Without fatty liver (mean ± SD), *n* = 80	With fatty liver (mean ± SD), *n* = 180
Age (years)	48.23 ± 10.70	47.99 ± 11.77	48.34 ± 10.24
Weight (kg)	64.611 ± 11.16	56.25 ± 6.29	68.33 ± 10.85
Height (cm)	157.931 ± 8.46	159.71 ± 7.84	157.27 ± 8.66
BMI	25.89 ± 3.86	22.12 ± 1.82	27.57 ± 3.30
Waist (cm)	89.96 ± 8.68	82.90 ± 3.79	93.09 ± 8.40
FBS (mg/dL)	166.08 ± 57.57	136.81 ± 35.90	179.09 ± 60.61
HDL (mg/dL)	47.10 ± 6.55	48.25 ± 5.93	46.59 ± 6.76
Triglyceride (mg/dL)	154.65 ± 50.25	137.50 ± 41.21	162.28 ± 52.09
AST (U/L)	36.05 ± 17.51	25.50 ± 8.23	40.74 ± 18.49
ALT (U/L)	35.94 ± 18.89	25.52 ± 8.34	40.57 ± 20.38
GGT (U/L)	45.78 ± 23.75	31.14 ± 12.06	52.29 ± 24.77
Insulin	12.81 ± 5.66	8.62 ± 2.88	14.68 ± 5.59
his	37.22 ± 4.36	33.62 ± 2.61	38.82 ± 4.02
FLI	50.04 ± 24.70	24.26 ± 10.03	61.50 ± 20.23
TyG	5.02 ± 0.27	4.88 ± 0.23	5.09 ± 0.26
VAI	2.53 ± 1.04	2.16 ± 0.70	2.70 ± 1.13
NAFLD-LFS	1.49 ± 1.39	0.52 ± 0.54	1.93 ± 1.43

BMI, body mass index; FBS, fasting blood sugar; HDL, high-density lipoproteins; AST, aspartate aminotransferase; ALT, alanine aminotransferase; GGT, gamma-glutamyl transferase; HSI, Hepatic Steatosis Index; FLI, Fatty Liver Index; TyG, Triglyceride and Glucose Index; VAI, Visceral Adiposity Index; NAFLD-LFS, NAFLD-Liver Fat Score.

Associations between the scale variables used to calculate the five liver fat indices and the ultrasonography-based diagnosis of fatty liver were determined using independent samples *t*-tests due to the significant variations in results between the individuals with a fatty liver and those without, and most of the scale variables showed significant correlations at the *p* < 0.001 level (**Table 3**).

**Table 3:** Associations between data collected and the liver ultrasonographic findings.

Imaging technique	Characteristics	*T* value	95% confidence interval		Significance (two-tailed)
			Lower	Upper	
Ultrasound scan	Weight (kg)	−9.285	−14.646	−9.521	< 0.001
	BMI	−13.86	−6.223	−4.675	< 0.001
	Waist (cm)	−10.38	−12.121	−8.254	<0.001
	FBS (mg/dL)	−5.80	−56.638	−27.926	<0.001
	Triglyceride (mg/dL)	−3.76	−37.749	−11.807	<0.001
	AST (U/L)	−7.06	−19.494	−10.995	<0.001
	ALT (U/L)	−6.36	−19.696	−10.388	<0.001
	GGT (U/L)	−7.26	−26.890	−15.413	<0.001
	Insulin	−9.17	−7.368	−4.761	<0.001

BMI, body mass index; FBS, fasting blood sugar; AST, aspartate aminotransferase; ALT, alanine aminotransferase; GGT, gamma-glutamyl transferase.

The significant variables were further statistically analyzed to achieve the objectives of the current study.

BMI classification based on the Asian population(16) was used for further analysis, and the current study population showed that irrespective of the fatty liver diagnosis, majority of the participants belong to the overweight category or above, which represented 76.5% (*n* = 199) of the population, of whom 24.2% (*n* = 63) were overweight, 33.1% (*n* = 86) were pre-obese, and 19.2% (*n* = 50) were obese; while the remaining participants were either normal weight (22.3%, *n* = 58) or underweight (1.2%, *n* = 3).

Among the patients diagnosed with fatty liver, 3.9% (*n* = 7) were of normal BMI, 22.2% (*n* = 40) had an overweight BMI, 46.1% (*n* = 83) belonged to the pre-obese category, while the remaining 27.8% (*n* = 50) belonged to the obese BMI category. The association between BMI and liver ultrasonography findings showed significance at *p* < 0.001.

The waist circumference was also statistically analyzed based on both the diagnosis of fatty liver and gender to aid in the data analysis process. Based on the classification related to fatty liver, it revealed that the mean waist circumference of the fatty liver diagnosed population was 93.9 ± 8.40 cm, which is considered higher than the recommended mean circumference for both men and women. Considering the gender-related classification, it showed that the mean waist circumference of the female participants in the study was higher (90.09 ± 8.74 cm; range: 77–111 cm) irrespective of the fatty liver diagnosis, while the male participants recorded a mean waist circumference of 89.68 ± 8.61 cm; range: 76–111 cm). The waist circumference was statistically significant against liver ultrasonography results (*p* < 0.001).

The three liver enzymes used in the current study were further classified based on their respective reference ranges to aid in the statistical analysis. Based on the results obtained, majority of the study population had normal levels of the liver enzymes irrespective of the fatty liver diagnosis. Of the total study population, 73.1% had normal AST levels, and 62.8% of those diagnosed with fatty liver through ultrasonography also showed normal AST levels while only 37.2% showed higher AST levels.

Similarly, 67.7% of the total population had normal ALT levels and only 43.8% of participants diagnosed with a fatty liver showed higher ALT levels while 56.1% of patients diagnosed with a fatty liver obtained normal ALT levels.

Of the total population, 73.8% also showed normal GGT levels while 63.9% of participants diagnosed with a fatty liver also showed normal GGT levels and only 36.1% of participants diagnosed with a fatty liver showed higher GGT levels. In contrast, majority of the participants without fatty liver showed normal liver enzyme levels where each percentage was over 90%.

The association of the biochemical investigations in the study with the liver ultrasonography findings was also determined, and most of the variables had strong associations with liver ultrasonographic findings at *p* < 0.001.

Associations were determined between liver ultrasonographic findings and the five liver fat indices—HSI, FLI, TyG, VAI, and NAFLD-LFS—using independent samples *t*-test (**Table 4**).

**Table 4:** Associations between liver fat indices and liver ultrasonographic findings.

Imaging technique	Liver fat index	*T* value	95% confidence interval		Significance (two-tailed)
			Lower	Upper	
Ultrasound scan	HSI	−9.896	−5.93328	−3.96172	<0.001
	FLI	−16.481	−39.15743	−30.7973	<0.001
	TyG	−6.043	−0.27182	−0.13820	<0.01
	VAI	−4.914	−0.78341	−0.33499	<0.01
	NAFLD-LFS	−11.515	−1.65973	−1.17492	<0.001

HSI, Hepatic Steatosis Index; FLI, Fatty Liver Index; TyG, Triglyceride and Glucose Index; VAI, Visceral Adiposity Index; NAFLD-LFS, NAFLD-Liver Fat Score.

Based on the results, three of the five liver fat indices (FLI, HSI, and NAFLD-LFS) used in the current study show that there is a strong statistical significance (*p* < 0.001) against the liver ultrasonographic findings, while the other two indices (TyG and VAI) show a moderate significance against liver ultrasonographic findings (*p* < 0.01).

Since the associations were determined between categorical and scale variables, where the independent variable (ultrasound scan) is a categorical variable and the dependent variables (five liver fat indices) are scale variables, the η^2^ value (effect size) was used to determine the strength of association of the variables (**Table 5**).

**Table 5:** Effect size of the liver fat indices.

Imaging technique	Liver fat indices	Eta value	Eta squared value
Ultrasound scan	HSI	0.480	0.230
	FLI	0.638	0.407
	TyG	0.352	0.123
	VAI	0.239	0.057
	NAFLD-LFS	0.469	0.219

HSI, Hepatic Steatosis Index; FLI, Fatty Liver Index; TyG, Triglyceride and Glucose Index; VAI, Visceral Adiposity Index; NAFLD-LFS, NAFLD-Liver Fat Score. Bold values indicates strength of association of the variables.

Comparisons were made between the grade of fatty liver and the five liver fat indices to determine which grade is best represented from each index. The means of the liver fat indices were compared with the grade of fatty liver using *post-hoc* tests with analysis of variance (ANOVA). Based on the results, three indices (FLI, his, and NAFLD-LFS) showed that each mean was statistically significantly different across all the three ultrasound scan categories (no fatty liver, grade 1 fatty liver, and grade 2 or above fatty liver) used in the study. FLI showed the highest mean difference among the three indices considering the patient population without a fatty liver and the patients with grade 2 fatty liver or above (**Table 6**).

**Table 6:** Comparisons between liver fat indices and grade of fatty liver.

Liver ultrasonography		Liver fat index	Mean difference	95% confidence interval	Significance
No fatty liver	Grade 2 fatty liver or above	FLI	−45.382	(−50.502)–(−40.262)	<0.001
		HSI	−6.036	(−7.238)–(−4.835)	<0.001
		NAFLD-LFS	−1.713	(−2.114)–(−1.312)	<0.001

FLI, Fatty Liver Index; HSI, Hepatic Steatosis Index; NAFLD-LFS, NAFLD-Liver Fat Score.

To further aid the analysis, the ROC curve was used to determine the performance of each index against the liver ultrasonographic findings—presence of fatty liver or absence of fatty liver. It was used to obtain the sensitivity and the specificity of each index and determine which index would be most appropriate to predict the presence of fatty liver based on the current study findings (**Table 7**).

**Table 7:** Comparison of AUROC between the participants.

Liver fat indices	Area under the curve (AUROC)		
	Total	Male	Female
Hepatic Steatosis Index	0.858	0.867	0.892
Fatty Liver Index	0.947	0.919	0.961
Triglyceride and Glucose Index	0.707	0.583	0.769
Visceral Adiposity Index	0.670	0.594	0.712
NAFLD-Liver Fat Score	0.847	0.743	0.898

HSI [AUC = 0.858 (95% CI: 0.812 to 0.902)] was able to determine patients with and without a fatty liver at an optimal cutoff value of 37.0213, with a sensitivity of 68.3% and a specificity of 95.5%.

FLI [AUC = 0.947 (95% CI: 0.923 to 0.971)] was able to distinguish between patients with and without a fatty liver at an optimal cutoff value of 46.120, with a sensitivity of 75% and a specificity of 100%.

TyG [AUC = 0.707 (95% CI: 0.642 to 0.772)] was able to determine the presence or absence of fatty liver in the study population at an optimal cutoff of 4.9312, with a sensitivity and specificity of 71.7% and 61.3%, respectively.

VAI [AUC = 0.670 (95% CI: 0.602 to 0.739)] was able to distinguish individuals with a fatty liver with an optimal cutoff value of 2.4577, with a sensitivity of 58.3% and a specificity of 76.2%.

NAFLD-LFS [AUC = 0.847 (95% CI: 0.800 to 0.894)] was able to determine the presence or absence of fatty liver of the study participants at an optimal cutoff value of 0.9056, with a sensitivity of 76.1% and a specificity of 85%.

The AUC was obtained using the plotted ROC curves to determine the classification performance of each index, and the results were interpreted according to the respective guidelines (**Figure 1**).

**Figure 1:**
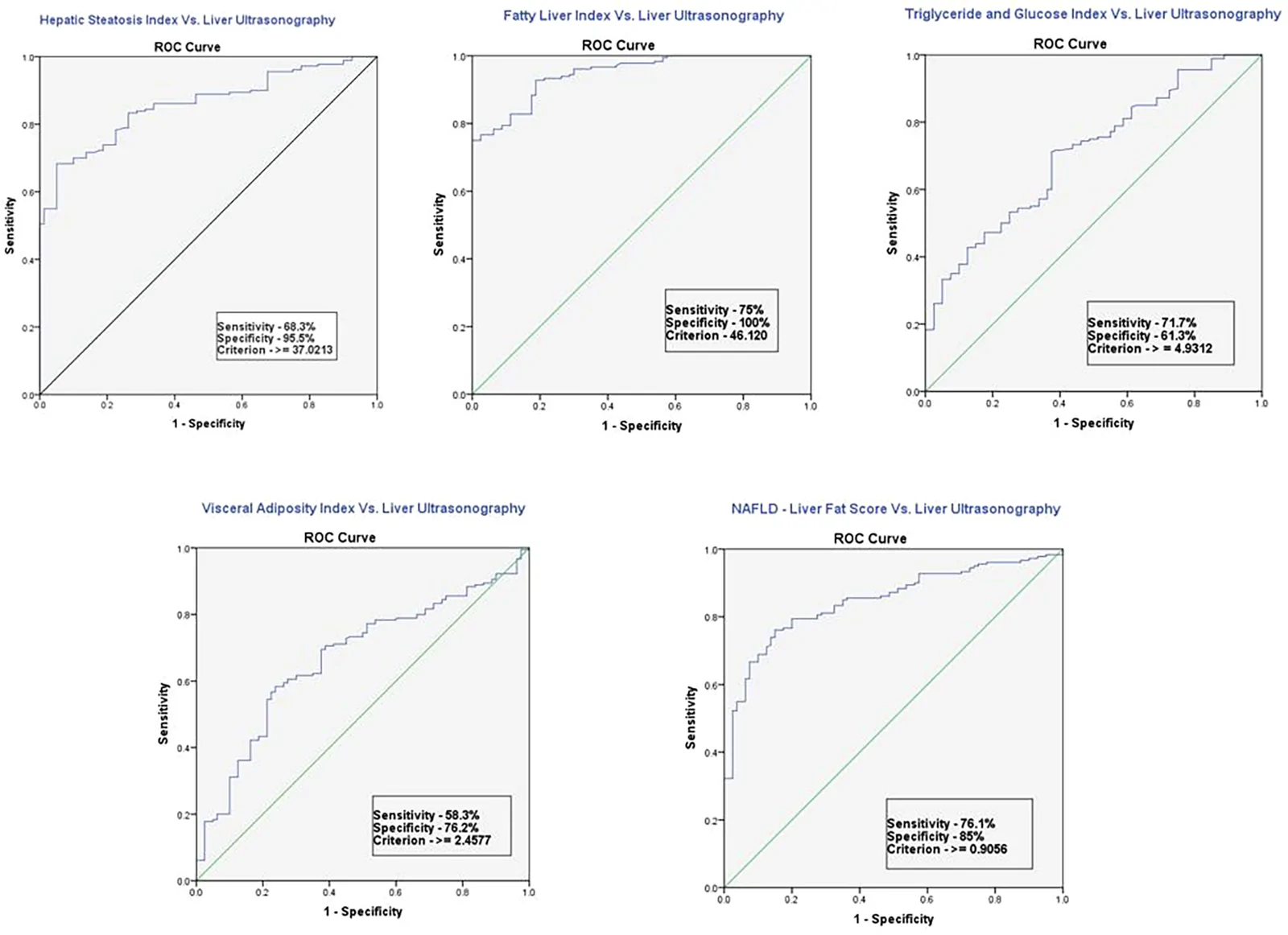
ROC curves for the five liver fat indices.

The AUROC analysis shown above for each index was based on the total study population of the current study. The AUROC analysis was also conducted to male and female participants separately to determine which liver fat index shows a strong association based on gender (**Table 7**), and all AUROC results were used in a graphical representation to obtain a clear understanding of the association between liver fat indices based on gender (**Figure 2**).

**Figure 2:**
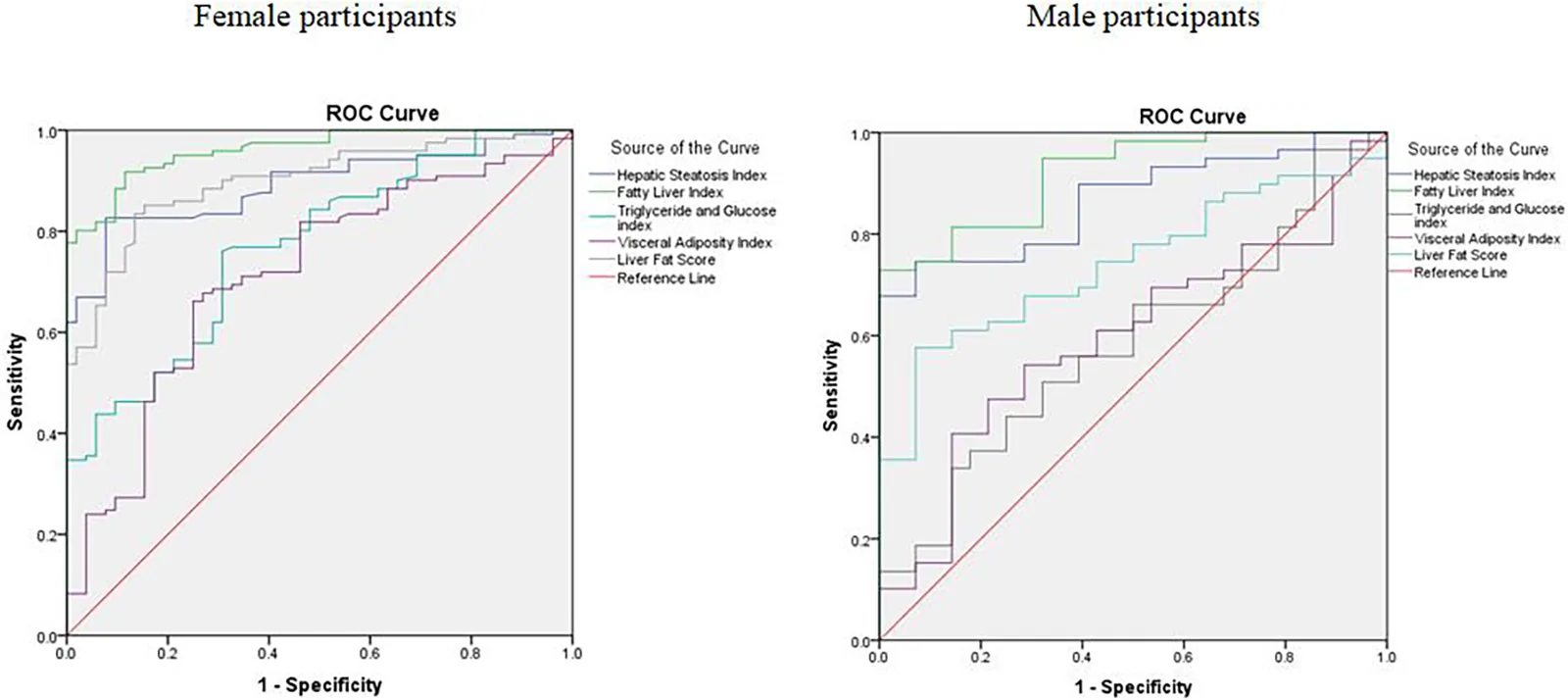
AUROC analysis based on gender.

Based on the results, a strong classification performance was observed among the female participants for HSI, FLI, and NAFLD-LFS even when compared with the results for the overall study population. The male participants showed a strong classification performance to only HSI and FLI based on the current study results.

Multivariable logistic regression was performed on the five indices separately to determine the independent predictive ability of fatty liver by each index. The aOR, 95% CI, and significance (*p*-value) were calculated (**Table 8**). Multicollinearity was evaluated on all variables, and it was ruled out since the VIFs of all variables fall below the threshold value of 5.0.

**Table 8:** Multivariable logistic regression on the indices.

Index	Adjusted odds ratio (aOR)	95% confidence interval (CI)	Significance (*p*-value)
Fatty Liver Index	1.144	1.090–1.201	<0.001
Hepatic Steatosis Index	1.366	1.157–1.614	<0.001
Visceral Adiposity Index	1.259	0.762–2.080	0.369
Triglyceride and Glucose Index	3.331	0.287–8.733	0.336
NAFLD Live Fat Score	2.031	0.959–4.299	0.064

The multivariable logistic regression analysis showed that FLI and HSI were independently associated with the presence of fatty liver. FLI was significantly associated with higher odds of fatty liver (aOR = 1.144, 95% CI: 1.090–1.201, *p* ≤ 0.001), while HSI also showed a significant association (aOR = 1.366, 95% CI: 1.157–1.614, *p* ≤ 0.001).

In contrast, VAI (aOR = 1.259, 95% CI: 0.762–2.080, *p* = 0.369), TyG (aOR = 3.331, 95% CI: 0.287–8.733, *p* = 0.336), and NAFLD-LFS (aOR = 2.031, 95% CI: 0.959–4.299, *p* = 0.064) were unable to retain the independent predictive ability of fatty liver based on the current study.

## 4 Discussion

The current study shows that the incidence of MASLD formerly known as NAFLD among newly diagnosed patients with T2DM was relatively high (69.2%). MASLD has become an emerging health threat in recent years, resulting in several complications as liver is considered an important organ responsible for metabolism in the human body. The prevalence of NAFLD among the Asian population has increased in the recent past, where a previous study showed a prevalence of NAFLD at 33% among the general population (18). Two previously conducted studies that were similar to the current study showed a prevalence of 82% and 63% among patients with T2DM based on liver ultrasonographic findings (19, 20).

Considering the results obtained in determining the association between the five liver fat indices and the liver ultrasonographic findings, there were three indices that showed highly significant correlations while the other two showed moderately significant correlations.

FLI, HSI, and NAFLD-LFS were the indices that showed strong significance, which were significant at the *p* < 0.001 level.

Based on the guidelines, η^2^ values were interpreted as follows: η^2^ <0.01 (negligible): there is virtually no association between the variables; η^2^ = 0.01–0.06 (small): the association is weak, but still detectable; η^2^ = 0.06–0.14 (medium): the association is moderately strong; and η^2^ > 0.14 (large): the association is strong; these indicate that a substantial amount of variance in the dependent variable can be explained by the independent variable, and the three indices FLI, HSI, and NAFLD-LFS all showed higher associations with η^2^ values of 0.407, 0.230, and 0.219, respectively.

The two remaining indices (TyG and VAI) showed moderately significant correlations at *p* < 0.01, and based on the η^2^ interpretation, Tyg showed a medium association (η^2^ = 0.123) and VAI showed a small association (η^2^ = 0.057).

The ROC curve was used to determine the performance of each index against the liver ultrasonographic findings, and it was used to obtain the respective sensitivities, specificities, and cutoff values for each index.

The highest performance was shown by FLI with an AUC of 0.947 and showed a sensitivity of 75% and specificity of 100%, and the optimal cutoff value to determine the presence of fatty liver was recorded as 46.120 based on the current study, which was also observed in a similar study where MASLD diagnostic criteria were considered (21). HSI showed a sensitivity of 68.3% and a specificity of 95.5% with an optimal cutoff value of 37.0213 to determine patients with or without a fatty liver. Similar results for AUC along with respective sensitivities and specificities for both FLI and HSI were found in several studies conducted on the same basis as the current study (22–24). A pilot study conducted based on the two indices also showed similar results based on the AUROC analysis (20).

When considering the other three indices used in the study, NAFLD-LFS also showed a strong performance with an AUC of 0.847 and a sensitivity and specificity of 76.1% and 85%, respectively. The index was able to predict the presence or absence of fatty liver with an optimal cutoff value of 0.9056 based on the current study population.

VAI and TyG were able to show a fair performance compared to the other three indices where VAI showed an AUC of 0.670 with a sensitivity and specificity of 58.3% and 76.2%, respectively, while TyG showed an AUC of 0.707 with a sensitivity and specificity of 71.7% and 61.3%, respectively.

The *post-hoc* tests with ANOVA were able to determine the significant differences of the means of liver fat indices across the grade of fatty liver used in the current study. The mean difference in FLI obtained between the patients with no fatty liver and patients with grade 2 fatty liver or above was the highest, and the results indicate that it is significantly different across the grade of fatty liver.

The AUROC analysis of each index based on gender showed that a strong classification performance was observed among the female participants for FLI, HIS, and NAFLD-LFS even when compared with the results for the overall study population, which aligned with a previously conducted study (21). The male participants showed a strong classification performance to only HSI and FLI based on the current study results.

The independent predictive ability determined by multivariable logistic regression analysis showed that both FLI and HSI were highly significant, while the remaining three indices, VAI, TyG, and NAFLD-LFS, did not maintain independent predictive significance in determining the presence or absence of fatty liver.

Based on the findings of the current study, both FLI (AUC = 0.947) and HSI (AUC = 0.858) showed strong classification performance based on the ROC analysis and independent predictive ability (aOR = 1.144, *p* ≤ 0.001; aOR = 1.366, *p* ≤ 0.001, respectively), which also corroborates with the findings of similar studies performed in relation to the indices (25, 26), whereas NAFLD-LFS, which showed a strong classification performance (AUC = 0.847), did not maintain a significant predictive ability according to the regression analysis (aOR = 2.031, *p* = 0.064) once it was adjusted to the non-embedded variables in the index’s formula.

Similarly, the two remaining indices, VAI and TyG, were also unable to retain the independent predictive significance though both indices showed moderate classification performance in the ROC analysis.

Liver fat indices mostly involve biochemical investigations that are frequently requested by clinicians and are routinely carried out in the Sri Lankan clinical setup including anthropometric measurements that can be collected from patients without any difficulty. The statistical significance of biochemical investigations like ALT, AST, and GGT with the liver ultrasonography findings shows that it can be used to predict the presence or absence of fatty liver as an alternative method, which was also found in previously conducted studies (7, 8). The current study findings show that the proposed liver fat indices can be used to predict the presence or absence of fatty liver when compared with the liver ultrasonographic findings. Among the five liver fat indices used in the current study, FLI and HSI can be considered as the strongest screening tools in determining the presence or absence of fatty liver due to their highly significant predictive ability as well as the strong classification performance based on the ROC analysis.

The gold standard in diagnosing MASLD/NAFLD is liver biopsy, but the routinely carried out investigation to determine fatty liver at present is liver ultrasonography due to the invasive procedures involved with performing liver biopsies. The ultrasound scan determines the presence or absence of fatty liver based on the echogenicity of the liver parenchymal cells, and the results may be affected by several factors like BMI over 40 and the presence of very low liver fat content (if <30%). FibroScan, which was introduced in recent years, measures fat accumulation and liver stiffness to determine the presence or absence of fatty liver with a higher sensitivity and specificity compared to ultrasound scan, though it is not readily available in most hospitals in the Sri Lankan clinical setup and is confined to specialized access in the government sector due to the limited resources. Therefore, it is not an easily accessible test procedure to all patients attending hospital clinics, as compared to ultrasound scan, which can be easily accessed by patients.

Moreover, patients who were recommended to undergo ultrasonography were mostly due to their elevated liver enzymes, and studies have shown that majority of the individuals diagnosed with a fatty liver had normal liver enzymes (27). Since the liver fat indices show significant associations with the liver ultrasonographic findings, it would provide an easy means of predicting the presence or absence of fatty liver since most of the biochemical investigations are routinely carried out for patients attending diabetic and endocrinology clinics in Sri Lanka and of assisting patients for further medical treatments and lifestyle modifications, leading to lesser complications.

### 4.1 Limitations

The study results may have been affected by variations in patient fasting conditions or nutritional status, which we could not monitor. Another parameter that may have affected the results is the data collected about the alcohol consumption of the participants, as they were self-reported by the participants themselves.

We were not able to use the liver histological samples as the diagnosis criteria for fatty liver even though the gold standard for the diagnosis of MASLD/NAFLD is liver biopsy since it is an invasive procedure and not performed routinely or FibroScan due to the limited resources available for this method in the hospital setup. The present study used a liver ultrasonography technique to determine the presence or absence of fatty liver and to grade fatty liver as it is the most common technique used in the available clinical setup.

### 4.2 Future recommendations

The liver fat indices could be evaluated against FibroScan in future studies to evaluate and validate the cutoff values, sensitivities, and specificities of the proposed five liver fat indices determined based on the liver ultrasonographic findings of the current study. In addition, it is recommended to use a higher sample size in a study that facilitates the development of a more suitable model in predicting the presence or absence of fatty liver considering further risk factors of fatty liver along with the factors used in the current study.

## 5 Conclusion

The current study showed that the proposed five liver fat indices have significant associations with the liver ultrasonographic findings. FLI showed the highest performance among the five indices and showed the strongest sensitivity and specificity based on the AUC analysis and also had the strongest independent predictive ability of fatty liver based on multivariable logistic regression analysis among the five liver fat indices. HSI also showed similar characteristics based on both statistical approaches while the remaining three indices—VAI, TyG, and NAFLD-LFS—were not significant to independently predict the presence of fatty liver even though they showed moderate performance in the AUC analysis. Based on the results of the current study, it can be concluded that both FLI and HSI can be used as a screening tool to predict MASLD/NAFLD among patients with T2DM.

## Data Availability

The raw data supporting the conclusions of this article will be made available by the authors, without undue reservation.
